# Morphology and Multigene Phylogeny Reveal Six New Species of *Micropsalliota* (Agaricaceae, Agaricales) from Southern China

**DOI:** 10.3390/jof12060419

**Published:** 2026-06-10

**Authors:** Shi-En Wang, Fei-Fei Song, Qing-Fu Huang, Cai-Xiang Huang, Ling-Hong Zeng, Zi-Yi Wang, Han-Bing Song, Dong-Mei Lin, Peng-Hu Liu

**Affiliations:** 1 College of Life Sciences, National Engineering Research Center of Juncao Technology, International College of Juncao Science, Fujian Agriculture and Forestry University, Fuzhou 350002, China; yinxin2022@126.com (S.-E.W.);; 2 College of Agriculture and Biological Science, Dali University, Dali 671003, China

**Keywords:** morphology, multi-gene phylogeny, taxonomy, new species, new record

## Abstract

*Micropsalliota* is a genus of small, slender agaric fungi predominantly distributed in tropical and subtropical regions. To explore the species diversity of *Micropsalliota* in southern China, we carried out both Maximum likelihood and Bayesian phylogenetic analyses based on multi-locus sequence data (ITS, nrLSU, *rpb2*, and *tef*1-α), combined with detailed morphological observations. Based on these analyses, six new species are described: *Micropsalliota alboglobulata*, *M. fuanensis*, *M. meilinensis*, *M. minutispora*, *M. pulvericlavata*, and *M. shenzhenensis*. In addition, *M. albosericea*, *M. gracilis*, and *M. purpureobrunneola* are reported for the first time in China. Detailed morphological descriptions, color photographs, line illustrations, and phylogenetic trees are provided.

## 1. Introduction

The genus *Micropsalliota* Höhn. was established in 1914 by the Austrian mycologist von Höhnel, with *Micropsalliota pseudovolvulata* Höhn. from Indonesia as the type species [1]. The genus was meant to include small, slender fungi that look like *Agaricus* but have violet spores [1,2]. It was subsequently revised by Heinemann (1956, 1976) and by Pegler and Rayner (1969) [3,4,5]. In 2016, Parra et al. showed that the genus *Allopsalliota* Nauta & Bas should be treated as a synonym of *Micropsalliota* [6]. *Micropsalliota* is placed in the family Agaricaceae [2,7,8,9], and has recently been classified within the subfamily Micropsalliotoideae by some researchers [10].

Progress in the molecular systematics of *Micropsalliota* began with Zhao et al. (2010) were the first to study the molecular phylogeny of this genus using ITS and nrLSU sequences, confirming that *Micropsalliota* is monophyletic, based on specimens from Thailand [2]. Subsequently, Yan et al. (2022) conducted the first Maximum likelihood and Bayesian analyses of a three-gene dataset (ITS, nrLSU, and *rpb2*), separating the genus into 6 major clades and 12 subclades, based on Chinese material [11]. More recently, Gao et al. (2024) analyzed four gene markers (ITS, nrLSU, *rpb2*, and *tef1-α*) from Chinese collections and revealed 11 major clades [12]. These molecular phylogenetic studies have provided an important foundation for further investigation of species diversity within the genus.

*Micropsalliota* species exhibit the following features: basidiomata usually small and slender, lamellae free, annulus membranous, spore print brown to dark brown, basidiospores with apical thickening, no germ pore, and dextrinoid, cheilocystidia often capitate or subcapitate, and the pileipellis a cutis with hyphae bearing incrusting pigments that turn olive-green in NH_4_OH solution [2,13,14]. Species of this genus are usually found in tropical and subtropical areas [15]. Their fruiting basidiomata are often small and easily overlooked, so the known species diversity remains underestimated [16]. In 2025, nine new species of *Micropsalliota* were reported worldwide, three of which are from China [16,17,18,19,20,21]. However, the species diversity of this genus still needs further exploration.

In China, since the first new species, *Micropsalliota pseudoglobocystis* Li Wei & R.L. Zhao, was reported [22], researchers have increasingly focused on the genus *Micropsalliota*. In this study, we examined specimens collected from southern China using both morphological observation and molecular phylogenetic analysis. We report six new species and three newly recorded species for China, which further enrich the known diversity of *Micropsalliota* in the country.

## 2. Materials and Methods

### 2.1. Specimens and Morphological Observations

The specimens for this study were collected from southern China, including Anhui Province, Chongqing Municipality, Fujian Province, Guangdong Province, Guangxi Zhuang Autonomous Region, Hunan Province, and Jiangsu Province. Fresh basidiomata were photographed in the field, and their macroscopic morphological characteristics were recorded promptly. The specimens were dried at 50 °C, stored in plastic self-sealing bags, and deposited in the fungarium of the Fujian Academy of Agricultural Sciences (FFAAS), China.

The macroscopic description and habitat details are based on detailed field records and photographs of fresh basidiomata. Color descriptions of fresh basidiomata follow the color system of Kornerup and Wanscher (1978) [23]. Microstructures were observed using dried specimens. The specimens were examined in 5% KOH solution or water, and stained with Congo red solution as needed. Melzer’s reagent was used to test whether the spores were amyloid. Observations were made using an NIB900 inverted biological microscope (Nexcope, Ningbo City, China). The morphological descriptions of the taxa followed the methods outlined in previous studies [2,11,12,24]. The following abbreviations are used in the descriptions: “(a) b–c (d)” is used to describe the size of basidiospores, where the “b–c” range represents 90% of the measured values, while “a” and “d” are the extreme values. “[Xav = e × f]” indicates the average size of basidiospores. “Q” refers to the length-to-width ratio of a single basidiospore from the side view, and “Qav” refers to the average value of “Q” across all specimens.

### 2.2. DNA Extraction, PCR Amplification, and Sequencing

Total genomic DNA was extracted following the method of Wang et al. (2025) [25]. The internal transcribed spacer (ITS) region, nuclear large subunit ribosomal DNA (nrLSU), the second largest subunit of RNA polymerase II (*rpb2*), and the translation elongation factor 1-α (*tef*1-α) were amplified using the primer pairs ITS1F/ITS4 [26,27], LR0R/LR7 [28], RPB2-6F/RPB2-7R [29], and EF1-983F/EF1-1567R [30], respectively. The composition of the reaction mixtures and the thermocycling conditions followed those described in Mou and Bau (2021) [31], and Gao et al. (2024) [12]. The PCR products were purified and sequenced by Sangon Biotech (Shanghai) Co., Ltd., Shanghai Municipality, China.

### 2.3. Phylogenetic Analyses

The chromatograms were checked in BioEdit v7.2.5 [32] to ensure that each sequence was of good quality. The final sequences were deposited in NCBI GenBank (Table 1 in bold). Reference sequences were selected based on previous studies and retrieved from NCBI GenBank, including ITS, nrLSU, *rpb2*, and *tef*1-α sequences of *Micropsalliota* [2,6,11,12,13,16,18,19,20,21,22]. Phylogenetic trees were reconstructed using the newly obtained sequences along with these reference sequences, encompassing all described species of *Micropsalliota* for which sequences are available (Table 1). *Agaricus campestris* LAPAG370 was used as an outgroup [2]. The ITS, nrLSU, *rpb2*, and *tef*1-α sequences were aligned independently using the G-INS-i algorithm implemented in the online MAFFT tool (https://mafft.cbrc.jp/alignment/server/, accessed on 14–17 April 2026), with the maximum iterative refinement set to two [33]. The resulting alignments were manually adjusted and trimmed at both ends in MEGA 7 [34]. Subsequently, the four loci were concatenated into a multi-locus dataset (ITS + nrLSU + *rpb2* + *tef*1-α) using the “Concatenate Sequence” function in PhyloSuite v2 [35], with missing data treated as “?”.

Phylogenetic analyses were performed using PhyloSuite v2. Maximum likelihood (ML) phylogenetic trees were inferred using IQ-TREE3 with 5000 ultrafast bootstraps, as well as the Shimodaira–Hasegawa-like approximate likelihood-ratio test [36,37]. Bayesian inference (BI) analyses were conducted using MrBayes 3.2.6 [38] under the partition model with two parallel runs, each with four Markov chain Monte Carlo (MCMC) chains. The runs were continued until the average standard deviation of split frequencies fell below 0.01, sampling every 100 generations, with the first 25% of sampled data discarded as burn-in. For both ML and BI analyses, the optimal substitution models were determined using ModelFinder [39]. The phylogenetic tree was visualized using FigTree v1.4.3 (http://tree.bio.ed.ac.uk/software/figtree/, accessed on 3 March 2025) and edited using Adobe Illustrator 2021 (Adobe, San Jose, CA, USA). Bootstrap support (BS) values ≥ 70% and Bayesian posterior probability (PP) values ≥ 0.80 are indicated on branches (BS/PP) and were considered statistically significant.

## 3. Results

### 3.1. Phylogenetic Analyses

The final multi-locus dataset (ITS + nrLSU + *rpb2* + *tef*1-α) for phylogenetic analysis comprised 2989 characters, including 1–801 bp from ITS, 802–1772 bp from nrLSU, 1773–2414 bp from *rpb2*, and 2415–2989 bp from *tef*1-α. The alignment consisted of 163 sequences with 2996 columns, 1381 distinct patterns 857 parsimony-informative, 202 singleton sites, 1937 constant sites. In this study, a total of 75 new sequences were generated, including 45 ITS, 11 nrLSU, 10 *rpb2*, and 9 *tef*1-α sequences (Table 1). The alignment was submitted to Figshare (https://figshare.com/s/529b9c65cce18d4ad771, accessed on 28 April 2026).

**Figure 1:**
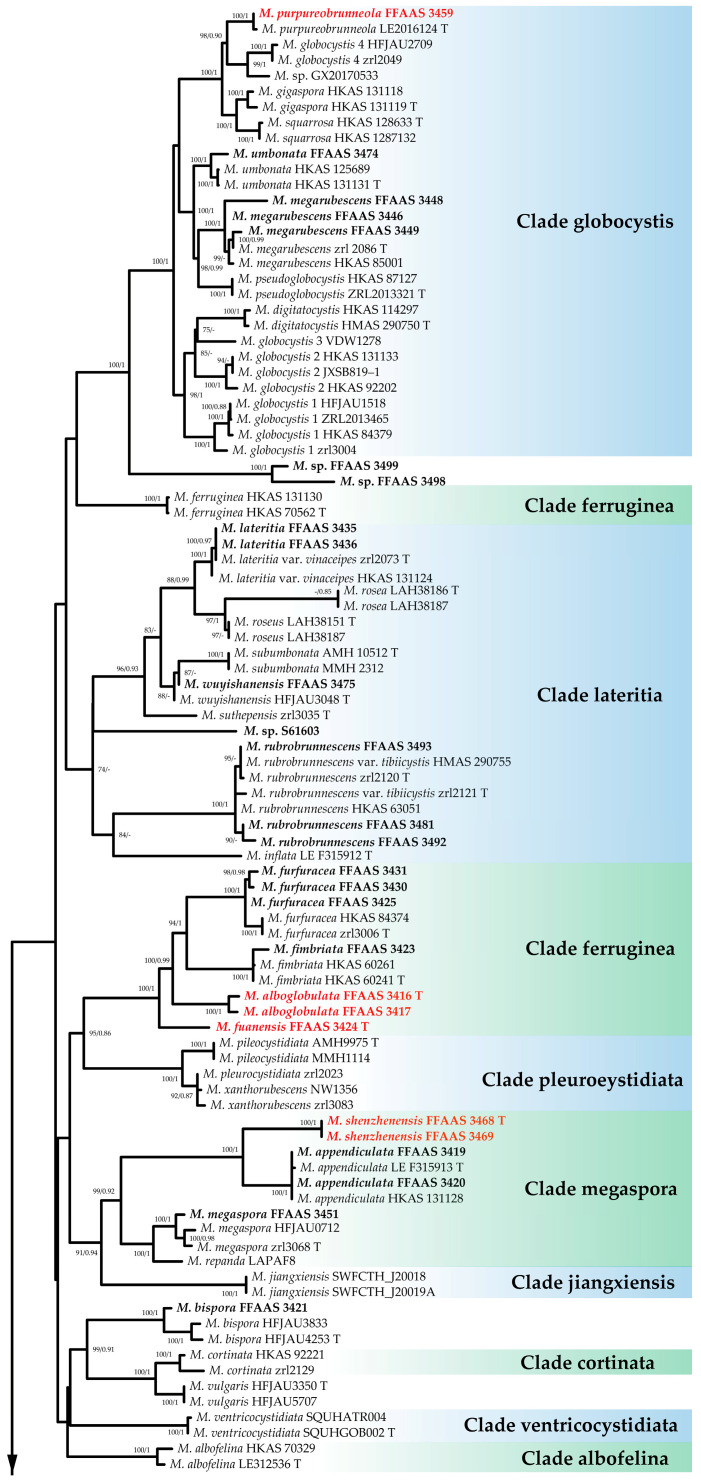

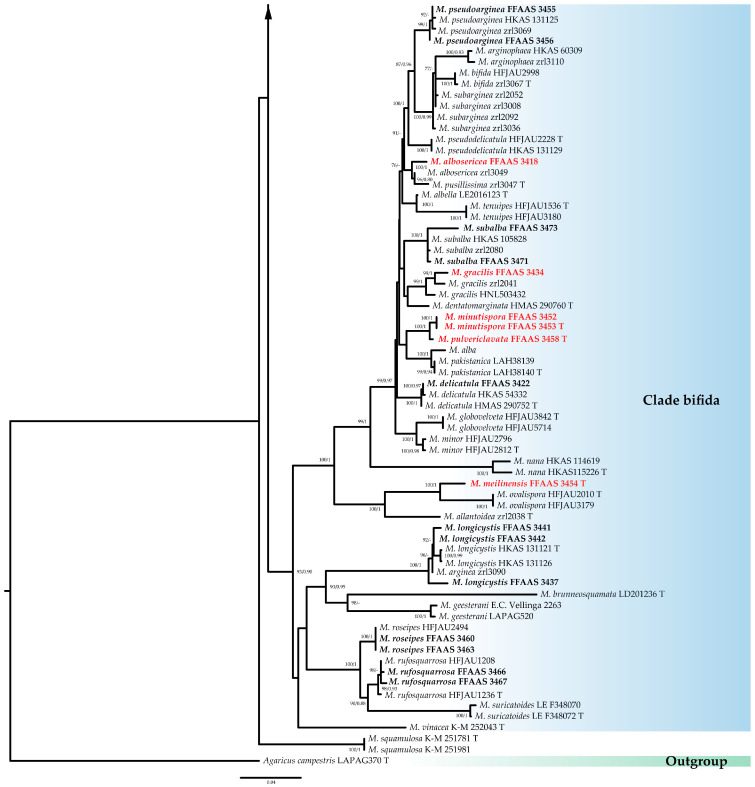
Phylogeny of *Micropsalliota* inferred from Bayesian and maximum-likelihood analyses of a multilocus dataset (ITS, nrLSU, *rpb2*, and *tef*1-α). Bold refers to the sequences produced from this study. Red font refers to the new species and newly recorded species. “T” refers to the type specimen.

The best-fit models according to the Bayesian Information Criterion (BIC) were as follows: for ML analysis, TVM+F+I+R3 for ITS, TIM+I+R4 for nrLSU, and TN+I+G4 for the combined *rpb2* and *tef*1-α dataset; for BI analysis, GTR+F+I+G4 for ITS, GTR+F+I+G4 for nrLSU, and HKY+I+G4 for the combined *rpb2* and *tef*1-α dataset. In the BI analysis, the average standard deviation of split frequencies fell below 0.01 after 5,100,000 generations. The tree topologies generated by BI and ML analyses were largely congruent. Therefore, only the ML tree is shown (Figure 1).

Our phylogenetic analysis (Figure 1) revealed a greater number of clades compared with the 11 major clades recognised by Gao et al. (2024) [12]. Clade bifida and Clade globocystis are relatively stable owing to their high species diversity, whereas the positions of other clades remain unstable. As noted by Yan (2025), the issue of clade stability in *Micropsalliota* remains incompletely resolved; however, with the discovery of additional taxa, more strongly supported clades are likely to emerge in future phylogenetic reconstructions [19]. Indeed, new clades have already been observed in previous phylogenetic analyses [16,21]. Overall, the *Micropsalliota* species included in the present study received support.

In the present phylogenetic analysis, the six newly described species were distributed across three distinct major clades. Within Clade bifida, *M. minutispora* and *M. pulvericlavata* formed a sister group with maximal statistical support (BS/PP = 100/1), while *M. meilinensis* and *M. ovalispora* likewise constituted a strongly supported sister pair (BS/PP = 100/1). Clade ferruginea comprised a fully supported lineage (BS/PP = 100/1) that included *M. alboglobulata*, *M. fimbriata*, *M. fuanensis*, and *M. furfuracea*; within this clade, *M. alboglobulata* and *M. fuanensis* each formed an independent evolutionary lineage. Additionally, *M. shenzhenensis* and *M. appendiculata* are sister groups in Clade megaspora (BS/PP = 100/1). The three newly recorded species for China—*M. albosericea*, *M. gracilis*, and *M. purpureobrunneola*—all clustered with their previously published conspecific sequences with high statistical support.

### 3.2. Taxonomy

***Micropsalliota alboglobulata*** S.E. Wang & P.H. Liu, sp. nov.

MycoBank No: MB863554

Figure 2

Etymology. *alboglobulata* (Latin), referring to the white fibrils on the pileus, which are composed of abundant globose cells.

Holotypus. China, Fujian Province, Longyan City, Liancheng County, Guanzhai Mountain, 20 August 2025, 116°46′56″ E, 25°42′48″ N, alt. 521 m, Shi-en Wang, E25082008 (FFAAS 3416).

Diagnosis. *Micropsalliota alboglobulata* is characterized by its white to reddish grey pileus covered with white fibrils, the absence of an annulus, and pileus squamules composed of abundant globose to ovoid and rarely short cylindrical cells.

Description. Pileus 2–6 mm in diameter, plano-convex to plane, surface dry, white (8A1) to reddish grey (8B2), with white (8A1) fibrils, gradually disappears with aging, appendiculate with annulus remnants. Context thin. Lamellae less than 1 mm broad, initially white (8A1), then reddish grey (8B2), later reddish brown (8E8), free, moderately distant, edge smooth, unequal, intercalated with numerous lamellulae. Stipe 3–17 × 0.2–0.8 mm, cylindrical, with slightly bulbous base, slender, hollow, white (8A1) to flesh (6B3), covered with white (8A1) fibrils, evanescent. Annulus unobserved.

Basidiospores (4.6)4.7–5.2(5.3) × (2.7)2.8–3.2(3.3) μm, [Xav = 4.9 × 2.9 μm], Q = 1.52–1.93, Qav = 1.69, ellipsoid to elongate-ellipsoid, light brown, with apical thickening, without germ pore, inamyloid. Basidia 9–14 × 5–6 μm, clavate, hyaline, 4(2)-spored, sterigmata 2–3 μm long. Cheilocystidia 21–38 × 3–11 μm, irregularly clavate to tibiiform, capitate with a long narrow neck, capitulum 4–6 μm in diameter, rarely subacute. Pleurocystidia absent. Pileus squamules composed of abundant globose to ovoid and rarely short cylindrical cells, 8–17 μm wide, hyaline, smooth, with some cells yellowish brown. Stipitipellis a cutis, hyphae 7–16 μm wide, light brown, slightly constricted at the septa, with thin hyphae 4–7 μm wide, terminal cells lanceolate.

Habitat and distribution. Gregarious or solitary on moss in August. Currently known only from Fujian Province, China.

Additional specimens examined. China, Fujian Province, Longyan City, 24 August 2025, Shi-en Wang, E25082409 (FFAAS 3417).

Notes. Morphologically, *Micropsalliota alboglobulata* shares a combination of features with *M. alba* Heinem. & Little Flower, *M. albosericea* Heinem. & Leelav., *M. delicatula* R.L. Zhao, J.Xin Li & M.Q. He, *M. minor* J.Q. Yan, and *M. pseudodelicatula* J. Q. Yan, including a white pileus generally less than 10 mm in diameter [2,11,40,41]. However, none of these species possess pileus squamules composed of abundant globose to ovoid and rarely short cylindrical cells.

It is worth noting that, within the genus *Micropsalliota*, pileus squamules (or pileipellis) composed of globose cells are rare, found only in a few species such as *M. globovelveta* J.Q. Yan & Y.T. Liu, *M. pruinosa* Heinem., and *M. pulverulenta* Heinem. & Leelav. However, *M. globovelveta* differs in having a reddish white to greyish rose pileus and a single annulus [16]. *Micropsalliota pruinosa* differs in having a finely pruinose-furfuraceous pileus, light brownish grey to fawn-rose colors, larger basidiospores (4.9–6.1 × 3.1–3.6 μm), and cheilocystidia that are capitate to subcapitate (16–20 × 4–9 μm) [42]. *Micropsalliota pulverulenta* differs in having a pruinose pileus surface that becomes dull vinaceous upon drying, larger basidiospores (4.5–5.2 × 3.5–4.1 μm, Q = 1.13–1.46), cheilocystidia that are cylindrical to ventricose-capitate (34.5–57.5 × 3.5–10 μm), and the presence of pileocystidia [43].

In the phylogenetic analysis (Figure 1), *M. alboglobulata* formed an independent lineage.

***Micropsalliota albosericea*** Heinem. & Leelav., Mycol. Res. 95(3): 341 (1991)

Figure 3

Description. Pileus 2–10 mm in diameter, conical when young, becoming plano-convex to plane with age, surface dry, white (8A1) to reddish grey (8B2), with white (8A1) fibrils, cuticle exceeding the denticulate margin. Context thin. Lamellae less than 2 mm broad, reddish grey (8B2) to reddish brown (8E8), free, subdistant, edge smooth, unequal, intercalated with numerous lamellulae. Stipe 6–17 × 0.6–0.9 mm, cylindrical, hollow, slender, white (8A1) to reddish grey (8B2), nearly smooth or with white (8A1) fibrils. Annulus single, superior or intermediate, membranous, white (8A1), pendent or percurrent, easily falling off.

Basidiospores (4.1)4.2–4.9(5.2) × (2.8)2.9–3.1(3.2) μm, [Xav = 4.6 × 3.0 μm], Q = 1.37–1.75, Qav = 1.53, ellipsoid to elongate-ellipsoid or cymbiform, light brown, with apical thickening, without germ pore, inamyloid. Basidia 10–14 × 5–6 μm, clavate, hyaline, 4-spored, sterigmata 1–2 μm long. Cheilocystidia 14–32 × 6–9 μm, clavate to ventricose-capitate or subcapitate, capitate on the top, capitulum 6–9 μm in diameter, hyaline, smooth. Pleurocystidia absent. Caulocystidia 12–21 × 5–7 μm, clavate or capitate. Pileus squamules composed of hyphae 4–9 μm wide, cylindrical, slightly constricted at the septa, hyaline, smooth. Stipitipellis a cutis, hyphae 7–15 μm wide, slightly constricted at the septa, light brown.

Habitat and distribution. Gregarious on moss in autumn. Currently known from China (Fujian), India (type locality), and Thailand.

Additional specimens examined. China, Fujian Province, Fuzhou City, Sandiejing Forest Park, 2 October 2025, Shi-en Wang, E2510221 (FFAAS 3418).

Notes. *Micropsalliota albosericea* was originally described from India, and subsequently reported from Thailand by Zhao et al., with molecular sequences generated [2,43]. Our specimens differ from both the Indian and Thai collections in that neither of the latter exhibits reddish tones on the pileus. Furthermore, the Indian collection has a silky smooth pileus and larger basidia (15–20 × 5–7.5 μm), while the collection from Thailand has lamellae that turn from white to orange-gray to brownish white. Our material is more similar to the Thailand collection. Notwithstanding these morphological differences, our sequences clustered with zrl3049 from Thailand in the phylogenetic analysis (Figure 1). Therefore, we identify the Chinese specimens as *M*. *albosericea*.

It is worth noting that caulocystidia were reported in the original description of *M*. *albosericea* from India but were not described in the Thailand collection. In the Chinese specimens, we observed caulocystidia similar to those from the Indian material, although they are not particularly prominent (clavate to capitate, 12–21 × 5–7 μm). These cells more closely resemble the terminal cells of the fibrils covering the stipe surface. Nevertheless, following the original description of the species, we still describe them as caulocystidia.

***Micropsalliota fuanensis*** S.E. Wang & P.H. Liu, sp. nov.

MycoBank No: MB863555

Figure 4

Etymology. *fuanensis* (Latin), referring to Fu’an City, the type locality of the holotype.

Holotypus. China, Fujian Province, Fu’an City, Pengjiayang Village, 27 July 2025, 119°36′29″ E, 27°04′50″ N, alt. 400 m, Deng Li, FACZ0191 (FFAAS 3424).

Diagnosis. *Micropsalliota fuanensis* is characterized by its pileus covered with reddish brown to brownish black squamules, small ellipsoid or cymbiform basidiospores (4.1–4.5 × 2.7–2.9 μm), and hyphoid to subclavate cheilocystidia that are often branched or forked.

Description. Pileus 5–8 mm in diameter, plano-convex to plane, surface dry, white (8A1) to pale grey (8B1), with reddish brown (8E8) to brownish black (8F8) fibrils aggregating into squamules, dense at the center, sparser toward the margin, appendiculate with annulus remnants. Context less than 1.0 mm thick. Lamellae 1–2 mm broad, pale gray (8B1), free, subdistant, edge smooth to serrate, unequal, intercalated with numerous lamellulae. Stipe 12–14 × 0.5–1 mm, cylindrical, hollow, pale gray (8B1) to brownish black (8F8), covered with white (8A1) tomentose fibrils. Annulus single, membranous, in the upper third of the stipe, white (8A1), easily falling off.

Basidiospores (4.0)4.1–4.5(4.6) × (2.6)2.7–2.9(3.0) μm, [Xav = 4.3 × 2.8 μm], Q = 1.41–1.64, Qav = 1.54, ellipsoid or cymbiform, light brown, with apical thickening, without germ pore, inamyloid. Basidia 8–12 × 4–6 μm, clavate, hyaline, 4(2)-spored, sterigmata 1–3 μm long. Cheilocystidia 17–44 × 4–7 μm, hyphoid or subclavate, tortuous, often branched or forked, apex obtuse or subcapitate, smooth, hyaline. Pleurocystidia absent. Pileus squamules composed of hyphae 6–16 μm wide, cylindrical, slightly constricted at septa, with brown membranous pigment. Stipitipellis a cutis, hyphae 5–11 μm wide, slightly constricted at the septa, light brown, with thin hyphae 3–7 μm wide.

Habitat and distribution. Gregarious or clustered on moss in summer. Currently known only from Fujian Province, China.

Notes. In the phylogenetic analysis (Figure 1), *Micropsalliota fuanensis* formed an independent lineage. Morphologically, *M. fuanensis* is similar to *M. malabarensis* Heinem. & Little Flower, particularly in pileus color, cymbiform basidiospores, and cheilocystidia morphology. However, *M. malabarensis* differs in having a pileus covered with brown squamulose-echinate scales, larger basidiospores (4.6–6.1 × 3.2–3.8 μm), larger basidia (13–16 × 6–7 μm), and a solitary habit on soil (or scattered on grassy humid soil) [2,40]. Although *M. wuyishanensis* Yan shares cheilocystidia of the same shape as *M. fuanensis*, it differs in having a red pileus covered with deep red fibrils, a stipe covered with pastel red to dull red fibrils, larger basidiospores (5.0–6.5 × 3.0–4.0 μm, Xav = 5.8 × 3.4 μm), and larger basidia (13–20 × 5.0–7.5 μm) [11].

***Micropsalliota gracilis*** Heinem., Bull. Jard. Bot. natn. Belg. 50(1–2): 60 (1980)

Figure 5

Description. Pileus 15–16 mm in diameter, plano-convex to plane, surface dry, ruddy (9B5) to blood red (10C8) and violet brown (10E8), with squamulose to furfuraceous, appendiculate with annulus remnants. Context less than 1.0 mm thick. Lamellae 1–2 mm broad, reddish brown (8E8), free, crowded, edge smooth, unequal, intercalated with numerous lamellulae. Stipe 30–31 × 1–2 mm, cylindrical, hollow, slender, white (8A1) to grey (8D1), covered with white (8A1) tomentose fibrils. Annulus single, superior, membranous, white (8A1), persistent, edge entire, reflexed.

Basidiospores (4.8)4.9–5.5(5.7) × (2.8)2.9–3.4(3.9) μm, [Xav = 5.2 × 3.1 μm], Q = 1.38–1.91, Qav = 1.69, ellipsoid to elongate-ellipsoid or amygdaliform, light brown, with apical thickening, without germ pore, inamyloid. Basidia 10–15 × 5–7 μm, clavate, hyaline, 4(2)-spored, sterigmata mostly 2–3 μm long, occasionally extending to 4–9 μm. Cheilocystidia 26–66 × 4–10 μm, ventricose to tibiiform or lageniform, capitate or obtuse at the apex, with a long narrow neck, capitulum 4–8 μm in diameter, hyaline, smooth. Pleurocystidia absent. Pileus squamules composed of hyphae 4–11 μm wide, cylindrical, slightly constricted at the septa, reddish brown, with vacuolar pigments. Stipitipellis a cutis, hyphae 9–15 μm wide, slightly constricted at the septa, light brown, with thin hyphae 3–7 μm wide.

Habitat and distribution. Solitary on forest ground in summer. Currently known from China (Fujian), Laos, Singapore (type locality), and Thailand.

Additional specimens examined. China, Fujian Province, Longyan City, Liancheng County, Guanzhai Mountain, 20 August 2025, Shi-en Wang, E25082015 (FFAAS 3434).

Notes. *Micropsalliota gracilis* was originally described from Singapore and later rediscovered in Thailand by Zhao et al. (2010), who also provided sequence data [2,44]. In contrast, the Chinese specimen is characterized by having an annulus that is reflexed (rather than pendent), smaller basidiospores (the original description reports 5.7–6.7 × 3.3–3.8 μm, Q = 1.72), the presence of 2-spored basidia, less diverse cheilocystidia, and a solitary (rather than gregarious) habit. Despite these differences, in the phylogenetic analysis (Figure 1), our specimen sequences cluster with zrl2041 from Thailand and HNL503432 from Laos; therefore, the specimen is identified as *M. gracilis*.

In China, *M*. *gracilis* is similar to *M. lateritia* Heinem. and *M. wuyishanensis* Yan in having a reddish pileus and a single annulus. However, *M. lateritia* differs in having a uniformly pink to dirty pink or slightly vinaceous pileus, cheilocystidia clavate or lanceolate-ventricose (22–40 × 9–18 μm), and a gregarious habit on bare forest soil [44,45]. *Micropsalliota wuyishanensis* differs in having a red (10A6–10B7) campanulate pileus covered with deep red fibrils, a stipe with pastel red to dull red fibrils, larger basidiospores (5.0–6.5 × 3.0–4.0 μm, Xav = 5.8 × 3.4 μm), larger basidia (13–20 × 5.0–7.5 μm), and cheilocystidia that are hyphoid, tortuous or submoniliform, often branched or forked [11].

***Micropsalliota meilinensis*** S.E. Wang & P.H. Liu, sp. nov.

MycoBank No: MB863557

Figure 6

Etymology. *meilinensis* (Latin), referring to Meilin Mountain Park, Shenzhen City, the type locality of the holotype.

Holotypus. China, Guangdong Province, Shenzhen City, Meilin Mountain Park, 29 September 2025, 114°03′08″ E, 22°34′33″ N, alt. 362 m, Yong-mei Cheng, CC2592901 (FFAAS 3454).

Diagnosis. *Micropsalliota meilinensis* is characterized by its white to reddish grey pileus covered with ruddy to brownish red fibrils forming fine squamules, the absence of an annulus, and small basidiospores.

Description. Pileus 11–18 mm in diameter, plano-convex to plane, margin recurved, surface dry, white (7A1) to reddish grey (7B1), with ruddy (9B5) to brownish red (10C8) fibrils aggregating into finely squamules, dense at the center, sparser toward the margin, appendiculate with annulus remnants. Context less than 1 mm thick. Lamellae 1–2 mm broad, reddish grey (9B3) to brownish black (8F8), free, moderately distant, edge smooth to serrate, unequal, intercalated with numerous lamellulae. Stipe 11–13 × 1–2 mm, cylindrical, hollow, flesh (6B3) to brownish black (8F8), covered with white (1A1) tomentose fibrils. Annulus unobserved.

Basidiospores (4.3)4.5–5.5(5.6) × (2.3)2.4–2.8(2.9) μm, [Xav = 4.9 × 2.7 μm], Q = 1.59–2.20, Qav = 1.81, ellipsoid to elongate-ellipsoid, light brown, with apical thickening, without germ pore, inamyloid. Basidia 11–17 × 5–7 μm, clavate, hyaline, 4(2)-spored, sterigmata 2–3 μm long. Cheilocystidia 27–42 × 4–8 μm, irregularly clavate or tibiiform, capitate with long narrow neck, capitulum 5–8 μm in diameter. Pleurocystidia absent. Pileus squamules composed of hyphae 5–17 μm wide, cylindrical, slightly constricted at septa, with light brown membranous pigment. Stipitipellis a cutis, hyphae 8–13 μm wide, slightly constricted at the septa, light brown, with thin hyphae 4–6 μm wide.

Habitat and distribution. Gregarious or scattered in broad-leaved forests on soil in autumn. Currently known only from Guangdong Province, China.

Notes. Morphologically, *Micropsalliota meilinensis* is similar to *M*. *cardinalis* Heinem. and *M. rufosquarrosa* J.Q. Yan. *Micropsalliota cardinalis* differs in having a violet-rose pileus covered with woolly to finely squamulose scales, larger basidiospores (5.2–6.8 × 3.1–3.6 μm), with a smaller Qav (Qav = 1.70), cheilocystidia 24–34 × 4–7 μm with a narrower capitulum (4.5–6 μm in diameter), and stipitipellis coating with hair-like cells [42]. *Micropsalliota rufosquarrosa* differs in having a pileus covered with red to brownish violet squarrose scales, a membranous annulus with a reddish margin, larger basidiospores (5.5–6.5 × 3.0–3.5 μm, Xav = 6.1 × 3.3 μm), and utriform cheilocystidia [11].

In the phylogenetic analysis (Figure 1), *M*. *meilinensis* groups with *M. allantoidea* R.L. Zhao, Desjardin, Soytong & K.D. Hyde and *M. ovalispora* J.Q. Yan. However, *M. allantoidea* differs in having a smaller pileus (5–10 mm in diameter), densely scaly grayish brown (7E3) pileus surface, stipe with a persistent membranous annulus, and basidiospores slightly larger (5.2–6.5 × 3–3.8 μm), with a smaller Qav (Qav = 1.73) [2]. *Micropsalliota ovalispora* differs in having a smaller pileus (5.0–7.0 mm in diameter), white (1A1) to orange–gray (6B2) pileus surface, lacking conspicuous fibrils or squamules, basidiospores smaller (4.0–5.0 × 2.5–3.0 μm, Xav = 4.5 × 2.7 μm) and oval to amygdaliform [11].

***Micropsalliota minutispora*** S.E. Wang & P.H. Liu, sp. nov.

MycoBank No: MB863556

Figure 7

Etymology. *minutispora* (Latin), referring to the very small basidiospores and the overall diminutive size of the basidiomata.

Holotypus. China, Fujian Province, Fuzhou City, Pingtan County, 23 May 2025, 119°48′15″ E, 25°30′34″ N, alt. 81 m, Shi-en Wang, E2552303 (FFAAS 3453).

Diagnosis. *Micropsalliota minutispora* is characterized by its small basidiomata (pileus 2–4 mm), white to pale brown pileus, and basidiospores measuring 3.7–4.3 × 2.6–2.7 μm (Xav = 4.1 × 2.6 μm, Qav = 1.55).

Description. Pileus 2–4 mm in diameter, hemispherical, expanding to plano-convex to plane, with obtuse umbo, surface dry, white (7A1), pale brown (7D3–5) at the center, with white (7A1) finely fibrillose, appendiculate with annulus remnants. Context less than 0.3 mm thick. Lamellae 0.6–1 mm broad, reddish grey (9B2) to brownish black (8F8), free, subdistant, unequal, intercalated with numerous lamellulae. Stipe 7–10 × 0.3–0.8 mm, cylindrical, hollow, white (6A1) to pale grey (6B1), covered with white (6A1) fibrils. Annulus single, membranous, persistent, superior, edge entire, white (1A1) to brownish black (8F8).

Basidiospores (3.5)3.7–4.3(4.4) × (2.4)2.6–2.7(2.9) μm, [Xav = 4.1 × 2.6 μm], Q = 1.37–1.71, Qav = 1.55, ellipsoid to elongate-ellipsoid or cymbiform, light brown, with apical thickening, without germ pore, inamyloid. Basidia 10–14 × 4–5 μm, clavate, hyaline, 4-spored, sterigmata 1–2 μm long. Cheilocystidia 11–29 × 4–8 μm, broadly clavate to clavate-capitate, rarely branched or irregular, or ellipsoid, with subcapitate apex, 4–6 μm in diameter, hyaline, smooth. Pleurocystidia absent. Pileus squamules composed of hyphae 3–7 μm wide, cylindrical, slightly constricted at septa, hyaline, yellowish brown, smooth. Stipitipellis a cutis, hyphae 7–15 μm wide, slightly constricted at the septa, light brown, with thin hyphae 3–6 μm wide.

Habitat and distribution. Solitary or scattered on sandy ground under *Araucaria cunninghamii* in spring. Currently known only from Fujian Province, China.

Additional specimens examined. China, Fujian Province, Fuzhou City, Pingtan County, 23 May 2025, Shi-en Wang, E2552302 (FFAAS 3452).

Notes. *Micropsalliota minutispora* is morphologically similar to *M. alba* Heinem. & Little Flower, *M. minor* J.Q. Yan, *M. pusillissima* R.L. Zhao, Desjardin, Soytong & K.D. Hyde, and *M. subalba* Heinem. & Little Flower, all of which share small basidiomata, a white pileus, and an annulus. However, *M. alba*, *M. minor*, and *M. subalba* have larger basidiospores and larger basidia [11,40]. The most similar species, *M. pusillissima*, also has larger basidiospores, and its cheilocystidia are broadly ventricose-capitate (20–30 × 6–11 μm, capitulum 7–11 μm in diameter), with a pendent or percurrent annulus [2].

In the phylogenetic analysis (Figure 1), *M*. *minutispora* and *M*. *pulvericlavata* are sister groups. The main morphological distinction is that *M. pulvericlavata* has a pileus with white (7A1) powdery-fibrillose and caulocystidia (clavate to clavate-capitate) (this study).

***Micropsalliota pulvericlavata*** S.E. Wang & P.H. Liu, sp. nov.

MycoBank No: MB863558

Figure 8

Etymology. *pulvericlavata* (Latin), referring to the white powdery-fibrillose pileus and clavate to clavate-capitate caulocystidia.

Holotypus. China, Chongqing Municipality, Shapingba District, 11 October 2020, 106°27′25″ E, 29°32′28″ N, alt. 263 m, Han-chen Wang, WHC2461 (FFAAS 3458).

Diagnosis. *Micropsalliota pulvericlavata* is characterized by its pileus with white powdery-fibrillose surface, brownish grey disc, stipe turning lake red to garnet brown when bruised, presence of rare caulocystidia (clavate to clavate-capitate).

Description. Pileus 3–11 mm in diameter, campanulate-conical, expanding to plano-convex to plane, with obtuse umbo, surface dry, sometimes fissurate, white (7A1), brownish grey (7C2–3) on the disc, with white (7A1) powdery-fibrillose, appendiculate with annulus remnants. Context thin. Lamellae 1–2 mm broad, reddish grey (9B3) to brownish black (8F8), free, subcrowded, unequal, intercalated with numerous lamellulae. Stipe 15–28 × 0.8–1.3 mm, cylindrical, hollow, white (7A1), covered with white (7A1) fibrils, surface turning lake red (9C8) to garnet brown (9D8) when bruised. Annulus single, membranous, persistent, superior to median, reflexed, margin often serrate, white (7A1).

Basidiospores (4.1)4.3–4.7(4.9) × (2.5)2.6–3.0(3.1) μm, [Xav = 4.5 × 2.8 μm], Q = 1.41–1.77, Qav = 1.60, ellipsoid to elongate-ellipsoid or cymbiform, light brown, with apical thickening, without germ pore, inamyloid. Basidia 10–15 × 4–6 μm, clavate, hyaline, 4-spored, sterigmata 2–3 μm long. Cheilocystidia 13–35 × 5–12 μm, pyriform or broadly clavate to clavate or utriform, with subcapitate or capitate apex, 8–9 μm in diameter, hyaline, smooth. Pleurocystidia absent. Caulocystidia present but rare, clavate to clavate-capitate, 23–70 × 6–15 μm, hyaline, smooth. Pileus squamules composed of hyphae 5–17 μm wide, cylindrical, slightly constricted at the septa, hyaline, with yellowish incrusting pigments in some hyphae. Stipitipellis a cutis, hyphae 8–17 μm wide, slightly constricted at the septa, light brown.

Habitat and distribution. Gregarious or clustered on moss in autumn. Currently known only from Chongqing Municipality, China.

Notes. *Micropsalliota pulvericlavata* is morphologically similar to *M. alba* Heinem. & Little Flower, *M. minor* J.Q. Yan, *M. minutispora*, *M. pusillissima* R.L. Zhao, Desjardin, Soytong & K.D. Hyde, and *M. subalba* Heinem. & Little Flower, all of which share small basidiomata, a white to grayish-brown pileus, and a membranous annulus. However, *M. alba* has a pure white pileus, larger basidiospores (5.8–6.6 × 3.3–3.6 μm), and larger basidia (14–17 × 5.5–6.5 μm) [40]. *Micropsalliota minor* has larger basidiospores (5.5–6.0 × 3.0–3.7 μm, Qav = 1.50–1.82) and cheilocystidia that are tibiiform or lageniform [11]. *Micropsalliota minutispora* has a pileus with white (7A1), finely fibrillose and lacks caulocystidia (this study). *Micropsalliota pusillissima* has a smaller pileus (1–3 mm), cheilocystidia that are broadly ventricose-capitate (20–30 × 6–11 μm, capitulum 7–11 μm in diameter), and a pendent or percurrent annulus [2]. *Micropsalliota subalba* has a larger pileus (8–23 mm), a longer stipe (30 × 2 mm), larger basidiospores (5.6–6.5 × 3.5–4.1 μm), larger basidia (16–20 × 6–7.5 μm), and cheilocystidia that are narrowly lanceolate to broadly capitate [40].

Notably, in the phylogenetic analysis (Figure 1), *M. pulvericlavata* and *M. minutispora* form a sister group relationship. For morphological differences between them, see the description of *M. minutispora*.

***Micropsalliota purpureobrunneola*** M.Q. He & R.L. Zhao, in He, Hyde, Cheewangkoon & Zhao, Phytotaxa 453(2): 140 (2020)

Figure 9

Description. Pileus 18–27 mm in diameter, plano-convex to plane, with obtuse umbo, surface dry, white (11A1), with triangular or granular, erect scales, brownish violet (11D7) to violet brown (11F8), appendiculate with annulus remnants. Context less than 2 mm thick. Lamellae 1–3 mm broad, brownish grey (8C2) to reddish brown (8E8), free, crowded, edge smooth, unequal, intercalated with numerous lamellulae. Stipe 45–71 × 2–4 mm, cylindrical, hollow, slender, white (8A1) to grey (8D1), covered with white (8A1) fibrillose scales, surface turning yellow when bruised. Annulus single, superior, membranous, white (8A1), with violet brown (11F8) margin, persistent, edge entire, reflexed.

Basidiospores (5.8)5.9–6.8(7.0) × (3.4)3.5–3.8(4.0) μm, [Xav = 6.3 × 3.6 μm], Q = 1.65–1.86, Qav = 1.75, ellipsoid to elongate-ellipsoid, light brown, with apical thickening, without germ pore, inamyloid. Basidia 9–13 × 6–8 μm, clavate, hyaline, 4(2)-spored, sterigmata 1–3 μm long. Cheilocystidia 23–50 × 4–8 μm, narrowly clavate to clavate-capitate, some slightly with a capitate apex, capitulum 7–12 μm in diameter, hyaline, smooth. Pleurocystidia absent. Pileus squamules composed of hyphae 7–20 μm wide, cylindrical, slightly constricted at the septa, reddish brown, with membranous pigments. Stipitipellis a cutis, hyphae 11–16 μm wide, slightly constricted at the septa, light brown, with thin hyphae 8–13 μm wide, terminal cells lanceolate.

Habitat and distribution. Gregarious on forest ground in summer. Currently known from China (Guangdong), and Thailand (type locality).

Additional specimens examined. China, Guangdong Province, Shenzhen City, Lianhua Mountain Park, 21 August 2025, Yong-mei Cheng, CC2508212 (FFAAS 3459).

Notes. *Micropsalliota purpureobrunneola* was first described from Thailand in 2020 [13]. The Chinese specimens differ from those from Thailand in having a larger pileus (18–27 mm vs. 5–12 mm in diameter), a longer stipe (45–71 mm vs. 16–22 mm), and smaller basidia (9–13 × 6–8 μm vs. 17.2–26.0 × 5.7–7.4 μm). Despite these size differences, the overall morphological features—particularly the distinctively colored erect scales, the yellowing upon bruising, and the annulus with a violet-brown margin—are consistent with *M. purpureobrunneola*. Moreover, in the phylogenetic analysis (Figure 1), our specimen sequences cluster with LE2016124 (holotype) from Thailand. Therefore, we identify the Chinese specimens as *M*. *purpureobrunneola*.

The Chinese specimens of *M. purpureobrunneola* are similar to *M. gigaspora* T. Gao & Z.W. Ge, *M. globocystis* Heinem., *M. pseudoglobocystis* Li Wei & R.L. Zhao, and *M. squarrosa* T. Gao & Z.W. Ge in having a whitish pileus covered with reddish brown or purplish scales, a stipe that turns yellow when bruised, and a single annulus. However, both *M. gigaspora* and *M. squarrosa* have a reddish brown pileus (*M. gigaspora*: 7E7; *M. squarrosa*: 6F4–6F6) and larger basidiospores (7–9 × 4–5.5 μm and 7–8.5 × 4.5–5.5 μm, respectively) [12]. Both *M. globocystis* and *M. pseudoglobocystis* have a larger pileus and longer stipe, but *M. globocystis* possesses larger basidiospores (5.6–7.4 × 3.7–4.2 μm), while *M. pseudoglobocystis* has smaller basidiospores (4.5–6 × 2.5–3.2 μm, Qav = 1.8) and larger basidia (15–20 × 5–7 μm) [22,44].

It is worth noting that *M. globocystis* represents a species complex, as this name corresponds to multiple lineages in the phylogenetic analysis [11,12].

***Micropsalliota shenzhenensis*** S.E. Wang & P.H. Liu, sp. nov.

MycoBank No: MB863559

Figure 10

Etymology. *shenzhenensis* (Latin), referring to Shenzhen City, the type locality of the holotype.

Holotypus. China, Guangdong Province, Shenzhen City, Lianhua Mountain Park, 1 July 2025, 114°05′27″ E, 22°34′54″ N, alt. 38 m, Yong-mei Cheng, CC2507011 (FFAAS 3468).

Diagnosis. *Micropsalliota shenzhenensis* is characterized by its pileus covered with fox to reddish brown scurfy fibrils, the absence of an annulus, and the presence of both cheilocystidia and pleurocystidia that are broadly clavate to broadly ventricose, often short-pedicellate.

Description. Pileus 4–13 mm in diameter, plano-convex to plane, surface dry, white (8A1) to greyish white (8B1), with fox (8D6–7) to reddish brown (8E8) scurfy fibrils, dense at the center, sparser toward the margin, appearing white (8A1), appendiculate with annulus remnants. Context thin. Lamellae 0.7–1.2 mm broad, white (8A1) to greyish white (8B1) to brownish black (8F8), free, subcrowded, edge smooth, unequal, intercalated with numerous lamellulae. Stipe 7–14 × 0.9–1.5 mm, cylindrical, hollow, white (8A1) to brownish black (8F8), with white (8A1) tomentose fibrils. Annulus unobserved.

Basidiospores (4.5)4.6–5.3(5.4) × (2.7)2.8–3.0(3.1) μm, [Xav = 5.0 × 2.9 μm], Q = 1.58–1.96, Qav = 1.71, ellipsoid to elongate-ellipsoid, light brown, with apical thickening, without germ pore, inamyloid. Basidia 10–14 × 5–6 μm, clavate, hyaline, 4(2)-spored, sterigmata 1–3 μm long. Cheilocystidia and pleurocystidia are common, similar, 18–38 × 5–13 μm, broadly clavate to broadly ventricose, rarely subcapitate, often short-pedicellate, hyaline. Pileus squamules composed of hyphae 5–21 μm wide, cylindrical, slightly constricted at the septa, with light brown membranous pigment, or with brown granular incrustations in cellular vacuoles in some hyphae. Stipitipellis a cutis, hyphae 7–15 μm wide, slightly constricted at the septa, light brown, with thin hyphae 4–9 μm wide, terminal cells lanceolate.

Habitat and distribution. Gregarious or clustered on forest ground in summer. Currently known only from Guangdong Province, China.

Additional specimens examined. China, Guangdong Province, Shenzhen City, Lianhua Mountain Park, 1 July 2025, Yong-mei Cheng, CC2507011–1 (FFAAS 3469).

Notes. In *Micropsalliota*, species with pleurocystidia are uncommon, including *M. appendiculata* D.D. Ivanova & O.V. Morozova, *M. digitatocystis* R.L. Zhao, J.Xin Li & M.Q. He, and *M. longicystis* T. Gao & Z.W. Ge. However, *M. appendiculata* differs in having a yellowish-white to brownish pileus covered with squamules, an annulus, larger basidiospores (5.5–7 × 3.5–4 μm, Xav = 6.15 × 3.85 μm), larger basidia (13–16 × 6–7 μm), and pleurocystidia that are broadly clavate to clavate-capitate (35–52 × 9–16 μm) [12]. *Micropsalliota digitatocystis* differs in having a reddish brown fibrillose-scaly pileus, an annulus, much larger basidiomata (pileus 16–74 mm in diameter, stipe 60–90 × 5–8 mm), larger basidiospores (5.8–7.4 × 4–4.6 μm), and pleurocystidia with a needle-like apex [41]. *Micropsalliota longicystis* differs in having an annulus, larger basidiospores (5–6 × 3–3.5 μm, Xav = 5.7 × 3.0 μm), larger basidia (14–19 × 5–8 μm), cheilocystidia that are clavate to clavate-capitate, and the presence of caulocystidia [12].

In the phylogenetic analysis (Figure 1), *M. shenzhenensis* and *M. appendiculata* are sister groups.

Notably, although *M. pleurocystidiata* Heinem. & Little Flower and *M. xanthorubescens* Heinem. also possess pleurocystidia, both species have pileus larger than 3 cm in diameter, and their basidiomata resemble those of *Agaricus* species [2,40,44]. Even *M. digitatocystis* resembles an *Agaricus* species [41]. Furthermore, *M. pleurocystidiata* and *M. xanthorubescens* are considered to represent the same species [2,11].

## 4. Discussion

In earlier studies, about 80 species of *Micropsalliota* were known worldwide, and 36 of them were found in China [16]. In this study, we report six new species and three newly recorded species for China. This brings the total number of *Micropsalliota* species known in China to 45. There is likely even more diversity of this genus in the country. Among our collections, *Micropsalliota* sp. FFAAS 3498 and *Micropsalliota* sp. FFAAS 3499 each had only one basidioma. Initially, we considered treating them as conspecific; however, clear macromorphological differences precluded this conclusion. Additional specimens are required to determine whether these collections represent two distinct species or morphological variants of a single taxon. Another collection, *Micropsalliota* sp. S61603, had several basidiomata, but they were too small and were accidentally lost during the study. Such loss is a frequent challenge when handling minute basidiomata.

These cases highlight a broader issue in fungal taxonomy. Many unnamed fungi found through DNA sequences cannot be formally named because the International Code of Nomenclature requires physical specimens for new species [46]. This has led scientists to consider whether we could give them temporary names based on DNA barcodes [47,48,49,50]. Some researchers even suggest that whole-genome data may soon become key evidence for separating species in fungal taxonomy, and “Species Delimitation 4.0”—which combines integrative taxonomy with artificial intelligence—may become the future standard [51,52].

Besides finding new species, we should also focus on revising previously described groups, especially those that are old and lack molecular data. For such species, we should add or update their molecular data. For groups with badly damaged specimens, we should collect new specimens and designate an epitype. This work is very important for current fungal taxonomy because it helps to solve many long-standing classification problems. For example, our analysis supports that *M. pleurocystidiata* and *M. xanthorubescens* are the same species. Also, *M. globocystis* represents a species complex. It is worth noting that earlier studies found that three sequences of *M. subarginea* from Thailand did not form a single clade, which suggested they might represent three different species [11]. In our study, however, they grouped together well. Similarly, *M. roseus* W. Akram, F. Maula, Saba, M. Asif [20] and *M. rosea* Akram, Maula, Saba & Asif [18], both from Pakistan, each have their own type sequences, but based on current evidence, they are likely the same species. Another illustrative case involves *M. longicystis* and *M. arginea* zrl3090: although they clustered together in our phylogenetic tree, the latter is represented solely by an nrLSU sequence and is not derived from type material; they are therefore provisionally retained as separate species pending further data. Local researchers possess inherent advantages in accessing and studying regional specimens, and these resources should be fully used to improve taxonomy.

Convergent evolution is a recurrent phenomenon in fungi. Within *Micropsalliota*, numerous species have independently evolved similar suites of traits, notably small basidiomata and a whitish pileus. Examples include *M*. *alboglobulata*, *M. delicatula*, *M. minor*, *M*. *minutispora* and *M. ovalispora*. In the field, accurate identification of these species based solely on morphological characters is challenging; thus, molecular data are necessary. However, phylogenetic analyses of *Micropsalliota* based solely on ITS sequences may lack sufficient resolution. To ensure taxonomic robustness, new species should be described using multi-locus analyses, especially by adding protein-coding genes such as *RPB2* and *tef*1-α. Notably, individual analysis of *RPB2* or *tef*1-α alone provided sufficient resolution to differentiate *Micropsalliota* species (data not shown).

Convergent morphological evolution is also evident between *Micropsalliota* and other genera. For example, the fruiting bodies of *M. digitatocystis*, *M. pleurocystidiata*, and *M. xanthorubescens* closely resemble those of *Agaricus* species. As noted previously [25], greater attention should be directed toward allied taxa that superficially resemble *Agaricus*. These examples highlight that convergent evolution can obscure generic boundaries in *Micropsalliota* and its allied taxa. Continued attention to allied genera such as *Leucoagaricus*, *Leucocoprinus*, and *Xanthagaricus* is warranted to avoid misidentification.

## Figures and Tables

**Figure 2:**
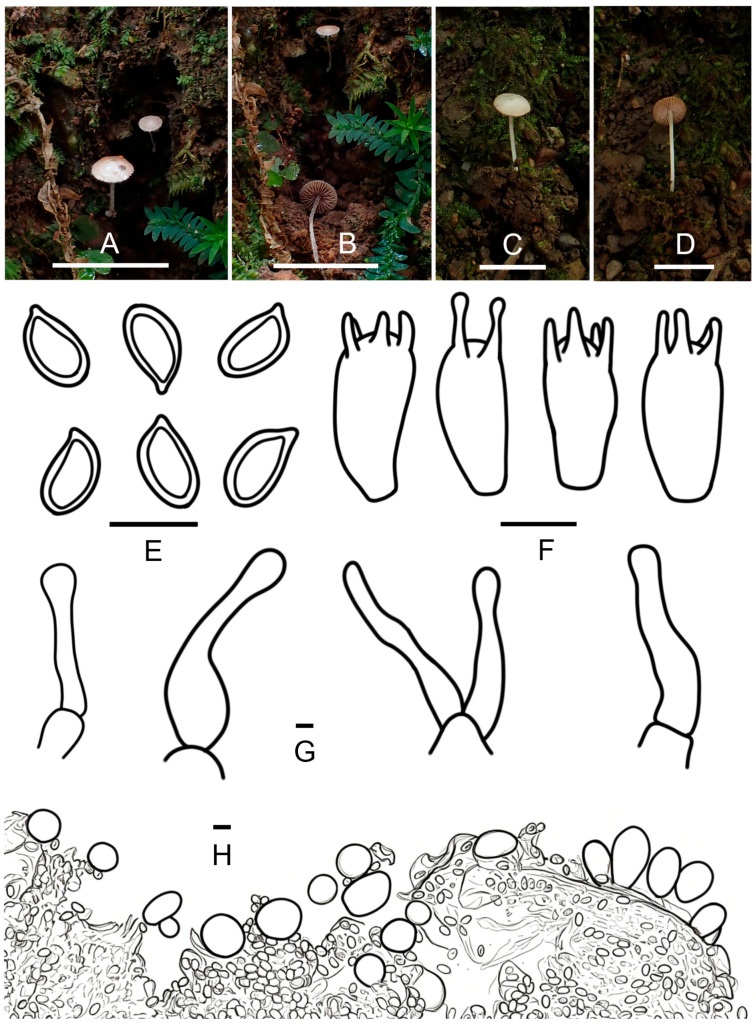
Morphological structures of *Micropsalliota alboglobulata*. (**A**–**D**) basidiomata, (**E**) basidiospores, (**F**) basidia, (**G**) cheilocystidia, (**H**) pileus squamules. Scale bars: 10 mm (**A**–**D**); 5 μm (**E**–**H**).

**Figure 3:**
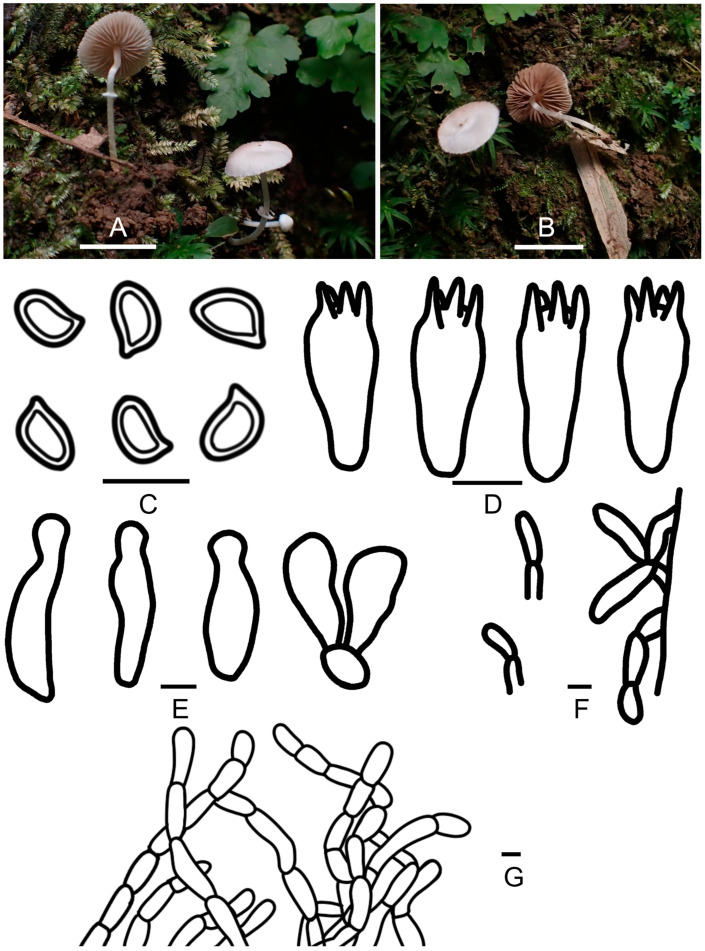
Morphological structures of *Micropsalliota albosericea*. (**A**,**B**) basidiomata, (**C**) basidiospores, (**D**) basidia, (**E**) cheilocystidia, (**F**) caulocystidia, (**G**) pileus squamules. Scale bars: 10 mm (**A**,**B**); 5 μm (**C**–**G**).

**Figure 4:**
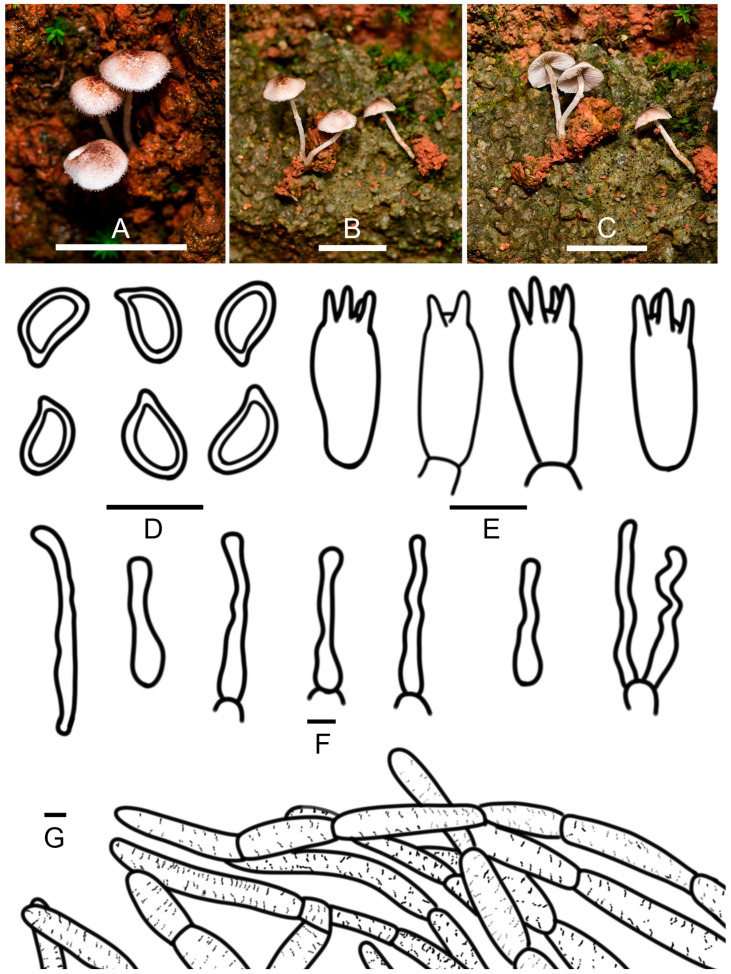
Morphological structures of *Micropsalliota fuanensis*. (**A**–**C**) basidiomata, (**D**) basidiospores, (**E**) basidia, (**F**) cheilocystidia, (**G**) pileus squamules. Scale bars: 10 mm (**A**–**C**); 5 μm (**D**–**G**).

**Figure 5:**
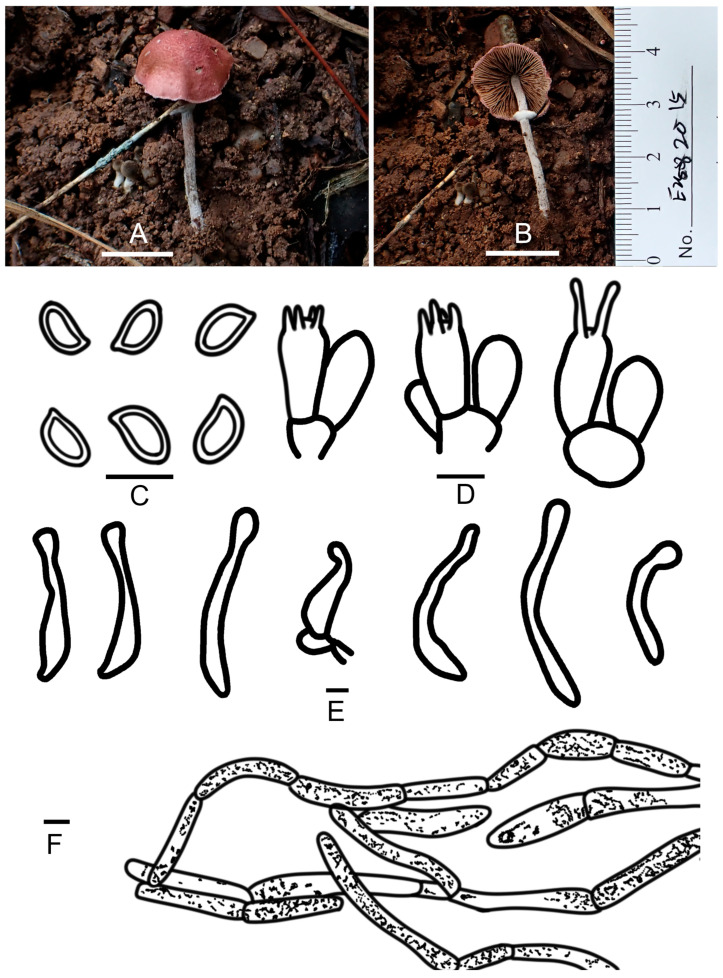
Morphological structures of *Micropsalliota gracilis*. (**A**,**B**) basidiomata, (**C**) basidiospores, (**D**) basidia, (**E**) cheilocystidia, (**F**) pileus squamules. Scale bars: 10 mm (**A**,**B**); 5 μm (**C**–**F**).

**Figure 6:**
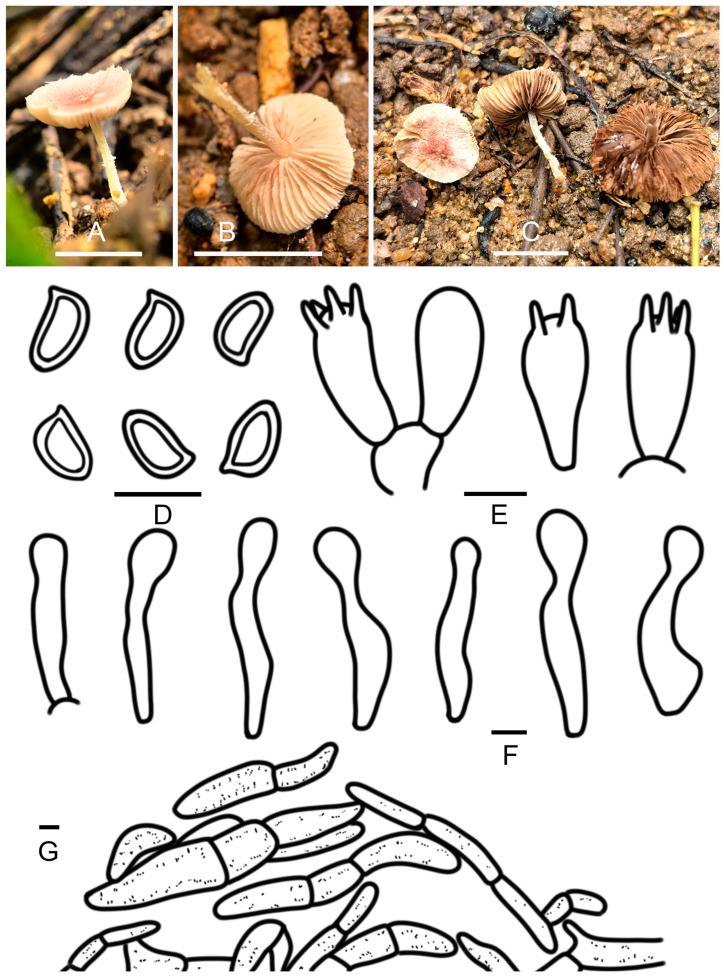
Morphological structures of *Micropsalliota meilinensis*. (**A**–**C**) basidiomata, (**D**) basidiospores, (**E**) basidia, (**F**) cheilocystidia, (**G**) pileus squamules. Scale bars: 10 mm (**A**–**C**); 5 μm (**D**–**G**).

**Figure 7:**
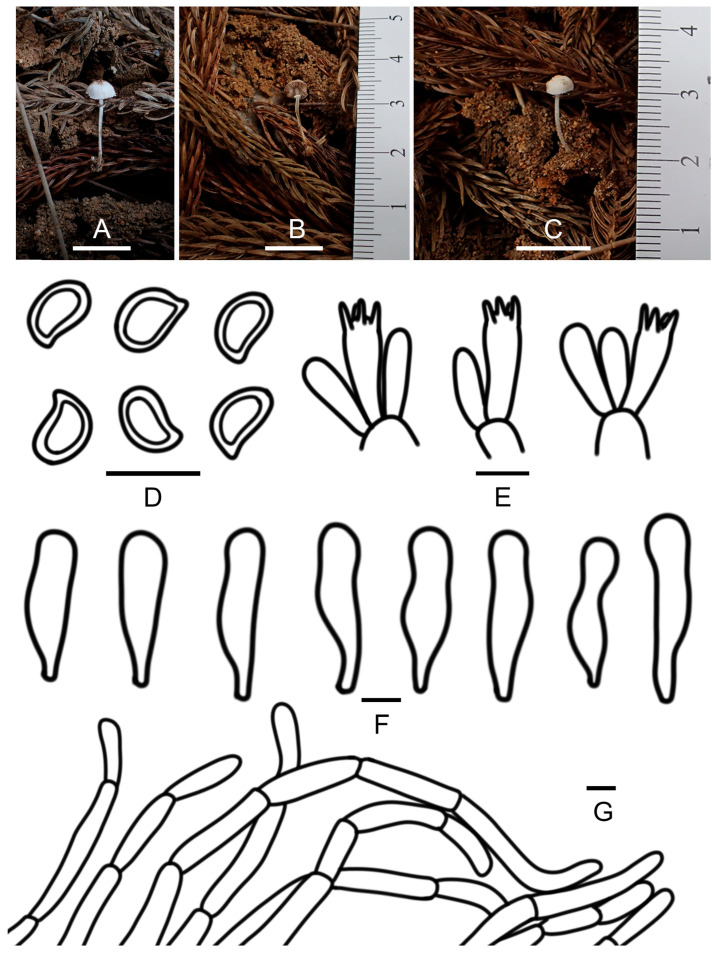
Morphological structures of *Micropsalliota minutispora*. (**A**–**C**) basidiomata, (**D**) basidiospores, (**E**) basidia, (**F**) cheilocystidia, (**G**) pileus squamules. Scale bars: 10 mm (**A**–**C**); 5 μm (**D**–**G**).

**Figure 8:**
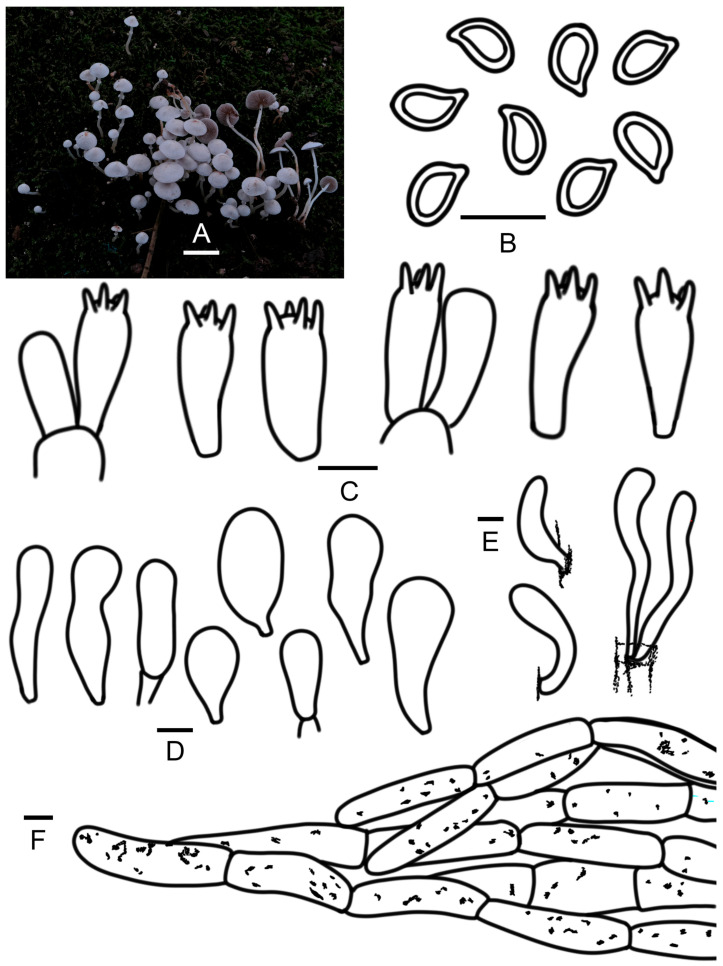
Morphological structures of *Micropsalliota pulvericlavata*. (**A**) basidiomata, (**B**) basidiospores, (**C**) basidia, (**D**) cheilocystidia, (**E**) caulocystidia, (**F**) pileus squamules. Scale bars: 10 mm (**A**); 5 μm (**B**–**F**).

**Figure 9:**
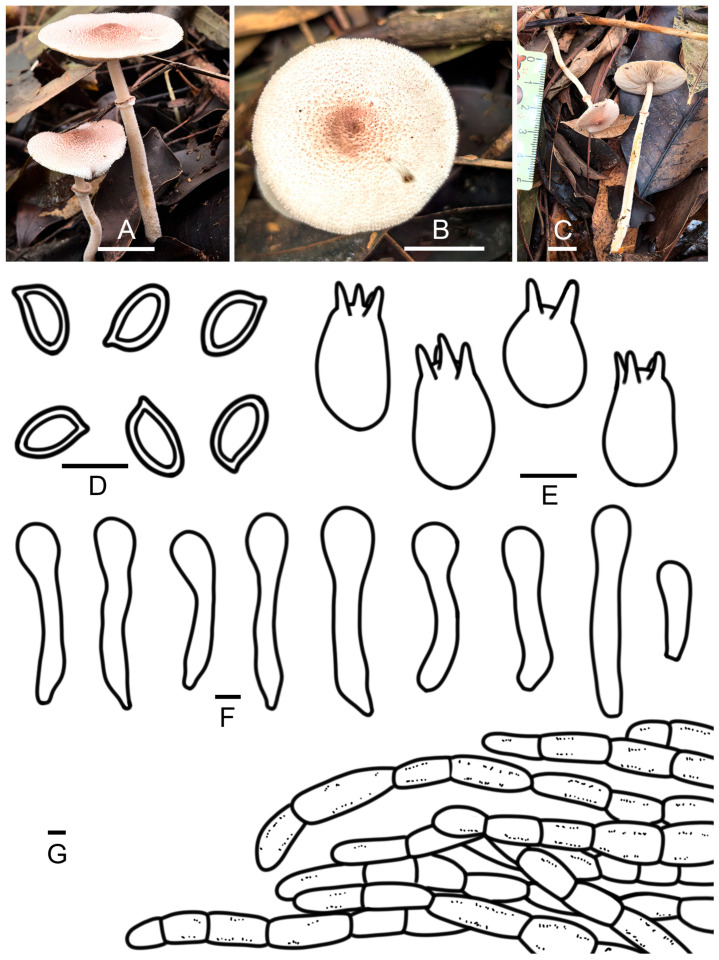
Morphological structures of *Micropsalliota purpureobrunneola*. (**A**–**C**) basidiomata, (**D**) basidiospores, (**E**) basidia, (**F**) cheilocystidia, (**G**) pileus squamules. Scale bars: 10 mm (**A**–**C**); 5 μm (**D**–**G**).

**Figure 10:**
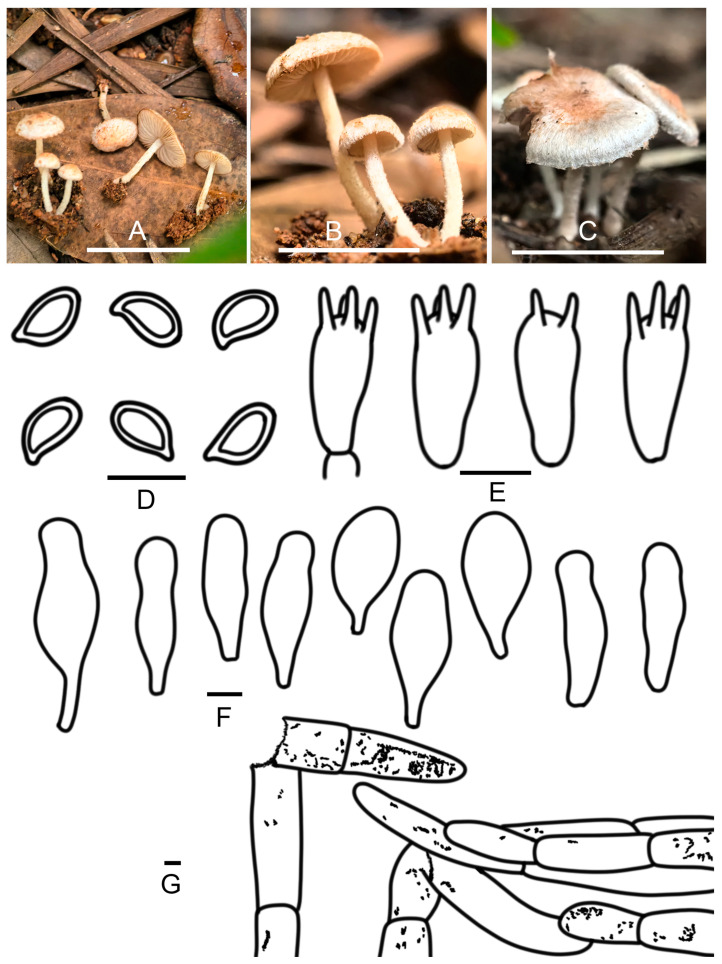
Morphological structures of *Micropsalliota shenzhenensis*. (**A**–**C**) basidiomata, (**D**) basidiospores, (**E**) basidia, (**F**) cheilocystidia/pleurocystidia, (**G**) pileus squamules. Scale bars: 10 mm (**A**–**C**); 5 μm (**D**–**G**).

**Table 1:** Sequences used in the phylogenetic analysis. Bold refers to the sequences produced from this study. Red font refers to the new species and newly recorded species. “T” refers to the type specimen. “—” means no relevant genetic information.

Taxon	Voucher	Locality	ITS	nrLSU	*rpb2*	*tef*1-α
*Micropsalliota alba*	—	India	EF069420	—	—	—
*M. albella*	LE2016123 T	Thailand	MN294514	MN294516	—	—
*M. albofelina*	HKAS 70329	China, Yunnan	OR799877	OR799922	OR962218	OR962180
*M. albofelina*	LE312536 T	Vietnam	OK257212	OK257209	—	—
***M. alboglobulata***	**FFAAS 3416 T**	**China, Fujian**	**PZ326316**	**—**	**—**	**PZ334552**
***M. alboglobulata***	**FFAAS 3417**	**China, Fujian**	**PZ326317**	**PZ326361**	**PZ334542**	**PZ334553**
*M. albosericea*	zrl3049	Thailand	HM436644	—	—	—
***M. albosericea***	**FFAAS 3418**	**China, Fujian**	**PZ326318**	**PZ326362**	**PZ334543**	**PZ334554**
*M. allantoidea*	zrl2038 T	Thailand	HM436648	HM436597	—	—
*M. appendiculata*	HKAS 131128	China, Yunnan	OR799910	OR799955	OR962246	OR962203
*M. appendiculata*	LE F315913 T	Vietnam	OR161109	OR161104	—	—
***M. appendiculata***	**FFAAS 3419**	**China, Guangxi**	**PZ326319**	**—**	**—**	**—**
***M. appendiculata***	**FFAAS 3420**	**China, Guangxi**	**PZ326320**	**—**	**—**	**—**
*M. arginea*	zrl3090	Thailand	—	HM436595	—	—
*M. arginophaea*	HKAS 60309	China, Hainan	OR799878	OR799923	OR962219	OR962208
*M. arginophaea*	zrl3110	Thailand	HM436617	HM436577	—	—
*M. bifida*	zrl3067 T	Thailand	HM436640	HM436591	—	—
*M. bifida*	HFJAU2998	China, Fujian	OM650272	OM650252	OM669858	—
*M. bispora*	HFJAU3833	China, Fujian	PQ345346	PQ345351	PQ358515	PQ358519
*M. bispora*	HFJAU4253 T	China, Fujian	PQ345347	PQ345352	PQ358516	PQ358520
***M. bispora***	**FFAAS 3421**	**China, Fujian**	**PZ326321**	**PZ326363**	**—**	**—**
*M. brunneosquamata*	LD201236 T	Thailand	KP316210	—	—	—
*M. cortinata*	HKAS 92221	China, Yunnan	OR799879	OR799924	OR962220	OR962183
*M. cortinata*	zrl2129	Thailand	HM436630	HM436593	—	—
*M. delicatula*	HKAS 54332	China, Yunnan	OR799880	OR799925	OR962221	OR962209
*M. delicatula*	HMAS 290752 T	China, Zhejiang	MT671229	—	—	—
***M. delicatula***	**FFAAS 3422**	**China, Fujian**	**PZ326322**	**—**	**—**	**—**
*M. dentatomarginata*	HMAS 290760 T	China, Guangxi	MT671228	MT671242	—	—
*M. digitatocystis*	HKAS 114297	China, Yunnan	OR799882	OR799927	OR962223	OR962184
*M. digitatocystis*	HMAS 290750 T	China, Yunnan	MT671239	MT671250	—	—
*M. ferruginea*	HKAS 70562 T	China, Yunnan	OR799884	OR799929	OR962225	OR962181
*M. ferruginea*	HKAS 131130	China, Yunnan	OR799885	OR799930	OR962226	OR962182
*M. fimbriata*	HKAS 60241 T	China, Hainan	OR799886	OR799931	OR962227	OR962198
*M. fimbriata*	HKAS 60261	China, Hainan	OR799887	OR799932	—	OR962199
***M. fimbriata***	**FFAAS 3423**	**China, Guangxi**	**PZ326323**	**—**	**—**	**—**
***M. fuanensis***	**FFAAS 3424 T**	**China, Fujian**	**PZ326324**	**PZ326364**	**—**	**—**
*M. furfuracea*	HKAS 84374	China, Yunnan	OR799888	OR799933	OR962228	OR962200
*M. furfuracea*	zrl3006 T	Thailand	HM436621	HM436603	—	—
***M. furfuracea***	**FFAAS 3425**	**China, Fujian**	**PZ326325**	**—**	**—**	**—**
***M. furfuracea***	**FFAAS 3430**	**China, Guangdong**	**PZ326326**	**—**	**—**	**—**
***M. furfuracea***	**FFAAS 3431**	**China, Guangdong**	**PZ326327**	**—**	**—**	**—**
*M. geesterani*	E.C. Vellinga 2263	The Netherlands	AF482857	AF482888	—	—
*M. geesterani*	LAPAG520	England	KM923965	KM923966	—	—
*M. gigaspora*	HKAS 131118	China, Yunnan	OR799890	OR799935	OR962230	—
*M. gigaspora*	HKAS 131119 T	China, Yunnan	OR799891	OR799936	OR962231	—
*M. globocystis* 1	HKAS 84379	China, Yunnan	OR799893	OR799938	OR962232	OR962187
*M. globocystis* 1	ZRL2013465	China	LT716024	KY418839	KY418991	KY419046
*M. globocystis* 1	HFJAU1518	China, Fujian	OM650277	OM650255	OM669852	—
*M. globocystis* 1	zrl3004	Thailand	HM436634	HM436605	—	—
*M. globocystis* 2	JXSB819–1	China, Jiangxi	MK402216	MK402224	—	—
*M. globocystis* 2	HKAS 92202	China, Yunnan	OR799894	OR799939	OR962233	OR962196
*M. globocystis* 2	HKAS 131133	China, Zhejiang	OR799895	OR799940	OR962234	OR962197
*M. globocystis* 3	VDW1278	South Africa	MT304640	—	—	—
*M. globocystis* 4	zrl2049	Thailand	HM436635	—	—	—
*M. globocystis* 4	HFJAU2709	China, Zhejiang	OM650278	OM650262	OM669856	—
*M. globovelveta*	HFJAU3842 T	China, Fujian	PV277734	PV269809	PV289604	PV289605
*M. globovelveta*	HFJAU5714	China, Fujian	PV277735	—	—	—
*M. gracilis*	zrl2041	Thailand	HM436647	HM436583	—	—
*M. gracilis*	HNL503432	Laos	MW192914	—	—	—
***M. gracilis***	**FFAAS 3434**	**China, Fujian**	**PZ326328**	**PZ326365**	**PZ334544**	**PZ334557**
*M. inflata*	LE F315912 T	Vietnam	OR161110	OR161106	—	—
*M. jiangxiensis*	SWFC_THJ20018	China, Jiangxi	ON117420	ON117438	—	—
*M. jiangxiensis*	SWFC_THJ20019A	China, Jiangxi	ON117421	ON117439	—	—
*M. lateritia*	FFAAS 3435	China, Fujian	PZ326329	—	—	—
***M. lateritia***	**FFAAS 3436**	**China, Guangxi**	**PZ326330**	**—**	**—**	**—**
*M. lateritia* var. *vinaceipes*	HKAS 131124	China, Yunnan	OR799896	OR799941	OR962235	OR962202
*M. lateritia* var. *vinaceipes*	zrl2073 T	Thailand	HM436631	—	—	—
*M. longicystis*	HKAS131121 T	China, Yunnan	OR799897	OR799942	OR962257	—
*M. longicystis*	HKAS131126	China, Yunnan	OR799898	OR799943	OR962258	—
***M. longicystis***	**FFAAS 3437**	**China, Jiangsu**	**PZ326331**	**—**	**—**	**—**
***M. longicystis***	**FFAAS 3441**	**China, Guangdong**	**PZ326332**	**—**	**—**	**—**
***M. longicystis***	**FFAAS 3442**	**China, Fujian**	**PZ326333**	**—**	**—**	**—**
*M. megarubescens*	HKAS 85001	China	OR799899	OR799944	OR962236	OR962188
*M. megarubescens*	zrl 2086 T	Thailand	HM436620	—	—	—
***M. megarubescens***	**FFAAS 3446**	**China, Fujian**	**PZ326334**	**—**	**—**	**—**
***M. megarubescens***	**FFAAS 3448**	**China, Guangdong**	**PZ326335**	**—**	**—**	**—**
***M. megarubescens***	**FFAAS 3449**	**China, Guangxi**	**PZ326336**	**—**	**—**	**—**
*M. megaspora*	zrl3068 T	Thailand	HM436624	—	—	—
*M. megaspora*	HFJAU0712	China, Jiangxi	OM650280	OM650256	OM669875	—
***M. megaspora***	**FFAAS 3451**	**China, Guangxi**	**PZ326337**	**—**	**—**	**—**
***M. meilinensis***	**FFAAS 3454 T**	**China, Guangdong**	**PZ326340**	**PZ326367**	**PZ334547**	**PZ334558**
*M. minor*	HFJAU2812 T	China, Zhejiang	OM650293	—	OM669864	—
*M. minor*	HFJAU2796	China, Zhejiang	OM650294	OM650266	OM669865	—
***M. minutispora***	**FFAAS 3452**	**China, Fujian**	**PZ326338**	**—**	**PZ334551**	**—**
***M. minutispora***	**FFAAS 3453 T**	**China, Fujian**	**PZ326339**	**PZ326366**	**PZ334545**	**PZ334555**
*M. nana*	HKAS 114619	China, Yunnan	OR799901	OR799946	OR962238	OR962216
*M. nana*	HKAS 115226 T	China, Yunnan	OR799902	OR799947	—	OR962217
*M. ovalispora*	HFJAU2010 T	China, Zhejiang	OM650295	OM650269	OM669866	—
*M. ovalispora*	HFJAU3179	China, Zhejiang	OM650296	—	—	—
*M. pakistanica*	LAH38139	Pakistan	PP357040	PP383879	—	—
*M. pakistanica*	LAH38140 T	Pakistan	PP357041	PP383880	—	—
*M. pileocystidiata*	AMH9975 T	India	MG917970	—	—	—
*M. pileocystidiata*	MMH1114	India	MZ598496	—	—	—
*M. pleurocystidiata*	zrl2023	Thailand	HM436636	—	—	—
*M. pseudoarginea*	HKAS 131125	China, Yunnan	OR799903	OR799948	OR962239	OR962212
*M. pseudoarginea*	zrl3069	Thailand	HM436643	—	—	—
***M. pseudoarginea***	**FFAAS 3455**	**China, Guangdong**	**PZ326341**	**—**	**—**	**—**
***M. pseudoarginea***	**FFAAS 3456**	**China, Fujian**	**PZ326342**	**—**	**—**	**—**
*M. pseudodelicatula*	HKAS 131129	China, Yunnan	OR799905	OR799950	OR962241	OR962213
*M. pseudodelicatula*	HFJAU2228 T	China, Zhejiang	OM650288	OM650264	OM669863	—
*M. pseudoglobocystis*	HKAS 87127	China, Sichuan	OR799908	OR799953	OR962244	OR962190
*M. pseudoglobocystis*	ZRL2013321 T	China, Yunnan	KM889913	—	—	—
***M. pulvericlavata***	**FFAAS 3458 T**	**China, Chongqing**	**PZ326343**	**PZ326368**	**PZ334546**	**PZ334556**
*M. purpureobrunneola*	LE2016124 T	Thailand	MN294513	MN294517	—	—
***M. purpureobrunneola***	**FFAAS 3459**	**China, Guangdong**	**PZ326344**	**PZ326369**	**PZ334548**	**PZ334559**
*M. pusillissima*	zrl3047 T	Thailand	HM436645	HM436594	—	—
*M. repanda*	LAPAF8	Togo	KP739805	KP739804	—	—
*M. rosea*	LAH38186 T	Pakistan	PQ870090	PQ871429	—	—
*M. rosea*	LAH38187	Pakistan	PQ871430	—	—	—
*M. roseipes*	HFJAU2494	China, Fujian	OM650297	OM650270	OM669870	—
***M. roseipes***	**FFAAS 3460**	**China, Hunan**	**PZ326345**	**—**	**—**	**—**
***M. roseipes***	**FFAAS 3463**	**China, Hunan**	**PZ326346**	**—**	**—**	**—**
*M. roseus*	LAH38151 T	Pakistan	PP377826	PP378507	—	—
*M. roseus*	LAH38187	Pakistan	PQ623105	PQ623107	—	—
*M. rubrobrunnescens*	HKAS 63051	China, Yunnan	OR799913	OR799957	OR962248	OR962205
*M. rubrobrunnescens*	zrl2120 T	Thailand	HM436628	HM436588	—	—
***M. rubrobrunnescens***	**FFAAS 3481**	**China, Fujian**	**PZ326347**	**—**	**—**	**—**
***M. rubrobrunnescens***	**FFAAS 3492**	**China, Fujian**	**PZ326348**	**—**	**—**	**—**
***M. rubrobrunnescens***	**FFAAS 3493**	**China, Guangxi**	**PZ326349**	**—**	**—**	**—**
*M. rubrobrunnescens* var. *tibiicystis*	zrl2121 T	Thailand	HM436629	HM436589	—	—
*M. rubrobrunnescens* var. *tibiicystis*	HMAS 290755	China, Guangxi	MT671231	—	MT671240	—
*M. rufosquarrosa*	HFJAU1236 T	China, Jiangxi	OM650292	OM650268	OM669869	—
*M. rufosquarrosa*	HFJAU1208	China, Jiangxi	OM650291	OM650267	OM669868	—
***M. rufosquarrosa***	**FFAAS 3466**	**China, Hunan**	**PZ326350**	**—**	**—**	**—**
***M. rufosquarrosa***	**FFAAS 3467**	**China, Hunan**	**PZ326351**	**—**	**—**	**—**
***M. shenzhenensis***	**FFAAS 3468 T**	**China, Guangdong**	**PZ326352**	**PZ326370**	**PZ334549**	**—**
***M. shenzhenensis***	**FFAAS 3469**	**China, Guangdong**	**PZ326353**	**—**	**—**	**—**
*M. squamulosa*	K-M 251781 T	Madagascar	PQ636525	—	—	—
*M. squamulosa*	K-M 251981	Madagascar	PQ636526	—	—	—
*M. squarrosa*	HKAS 128633 T	China, Yunnan	OR799915	OR799959	OR962250	—
*M. squarrosa*	HKAS 1287132	China, Yunnan	OR799916	OR799960	OR962251	—
*M. subalba*	HKAS 105828	China, Guangdong	OR799917	OR799961	—	OR962211
*M. subalba*	zrl2080	Thailand	HM436646	HM436596	—	—
***M. subalba***	**FFAAS 3471**	**China, Anhui**	**PZ326354**	**—**	**—**	**—**
***M. subalba***	**FFAAS 3473**	**China, Fujian**	**PZ326355**	**—**	**—**	**—**
*M. subarginea*	zrl2092	Thailand	HM436611	HM436574	—	—
*M. subarginea*	zrl2052	Thailand	HM436612	HM436573	—	—
*M. subarginea*	zrl3036	Thailand	HM436610	HM436575	—	—
*M. subarginea*	zrl3008	Thailand	—	HM436576	—	—
*M. subumbonata*	AMH 10512 T	India	PP837559	PQ576415	—	—
*M. subumbonata*	MMH 2312	India	PQ577838	PQ580740	—	—
*M. suricatoides*	LE F348070	Vietnam	OR161114	OR161107	—	—
*M. suricatoides*	LE F348072 T	Vietnam	OR161112	—	—	—
*M. suthepensis*	zrl3035 T	Thailand	—	HM436584	—	—
*M. tenuipes*	HFJAU1536 T	China, Fujian	OM650289	—	—	—
*M. tenuipes*	HFJAU3180	China, Fujian	OM650290	OM650265	—	—
*M. umbonata*	HKAS 125689	China, Yunnan	OR799918	OR799962	OR962252	OR962192
*M. umbonata*	HKAS 131131 T	China, Yunnan	OR799920	OR799964	OR962254	OR962194
***M. umbonata***	**FFAAS 3474**	**China, Guangdong**	**PZ326356**	**—**	**—**	**—**
*M. ventricocystidiata*	SQUHGOB002 T	Oman	OM397374	OM630414	—	—
*M. ventricocystidiata*	SQUHATR004	Oman	OM397373	OM630413	—	—
*M. vinacea*	K-M 252043 T	Madagascar	PQ636527	—	—	—
*M. vulgaris*	HFJAU3350 T	China, Zhejiang	PQ345344	PQ345349	PQ358513	PQ358517
*M. vulgaris*	HFJAU5707	China, Zhejiang	PQ345345	PQ345350	PQ358514	PQ358518
*M. wuyishanensis*	HAFJAU3048 T	China, Fujian	OM650298	—	OM669878	—
***M. wuyishanensis***	**FFAAS 3475**	**China, Fujian**	**PZ326357**	**—**	**—**	**—**
*M. xanthorubescens*	NW1356	Thailand	MW504965	—	—	—
*M. xanthorubescens*	zrl3083	Thailand	HM436638	HM436598	—	—
*M.* sp.	GX20170533	China, Guangxi	MT671232	MT671248	—	—
***M.*** **sp.**	**FFAAS 3498**	**China, Hunan**	**PZ326358**	**—**	**—**	**—**
***M.*** **sp.**	**FFAAS 3499**	**China, Guangxi**	**PZ326359**	**PZ326371**	**PZ334550**	**PZ334560**
***M.*** **sp.**	**S61603**	**China, Anhui**	**PZ326360**	**—**	**—**	**—**
*Agaricus campestris* (Outgroup)	LAPAG370 T	Spain	KM657927	KP739803	KT951556	KR006636

## Data Availability

All of the data that support the findings of this study are available in the main text, or in publicly accessible data repositories, as indicated in the text. The Chinese names of the newly described taxa are provided in a publicly accessible Figshare repository (https://figshare.com/s/529b9c65cce18d4ad771, accessed on 29 April 2026).

## Abbreviations

The following abbreviations are used in this manuscript:

BI	Bayesian inference
BS	Bootstrap support
FFAAS	Fungarium of the Fujian Academy of Agricultural Sciences
ITS	Internal transcribed spacer
KOH	Potassium hydroxide
ML	Maximum likelihood
nrLSU	Nuclear large subunit ribosomal DNA
NH4OH	Ammonium hydroxide
PP	Posterior probability
rpb2	Second largest subunit of RNA polymerase II
tef1-α	Translation elongation factor 1-α
